# Assessing the combined effects of climatic factors on spring wheat phenophase and grain yield in Inner Mongolia, China

**DOI:** 10.1371/journal.pone.0185690

**Published:** 2017-11-03

**Authors:** Junfang Zhao, Feiyu Pu, Yunpeng Li, Jingwen Xu, Ning Li, Yi Zhang, Jianping Guo, Zhihua Pan

**Affiliations:** 1 State Key Laboratory of Severe Weather, Chinese Academy of Meteorological Sciences, Beijing, China; 2 Resources College, Sichuan Agricultural University, Chengdu, PR China; 3 Inner Mongolia Ecology and Agrometeorology Center, Hohhot, China; 4 College of Resources and Environmental Sciences, China Agricultural University, Beijing, China; Murdoch University, AUSTRALIA

## Abstract

Understanding the regional relationships between climate change and crop production will benefit strategic decisions for future agricultural adaptation in China. In this study, the combined effects of climatic factors on spring wheat phenophase and grain yield over the past three decades in Inner Mongolia, China, were explored based on the daily climate variables from 1981–2014 and detailed observed data of spring wheat from 1981–2014. Inner Mongolia was divided into three different climate type regions, the eastern, central and western regions. The data were gathered from 10 representative agricultural meteorological experimental stations in Inner Mongolia and analysed with the Agricultural Production Systems Simulator (APSIM) model. First, the performance of the APSIM model in the spring wheat planting areas of Inner Mongolia was tested. Then, the key climatic factors limiting the phenophases and yield of spring wheat were identified. Finally, the responses of spring wheat phenophases and yield to climate change were further explored regionally. Our results revealed a general yield reduction of spring wheat in response to the pronounced climate warming from 1981 to 2014, with an average of 3564 kg·ha^-1^. The regional differences in yields were significant. The maximum potential yield of spring wheat was found in the western region. However, the minimum potential yield was found in the middle region. The air temperature and soil surface temperature were the optimum climatic factors that affected the key phenophases of spring wheat in Inner Mongolia. The influence of the average maximum temperature on the key phenophases of spring wheat was greater than the average minimum temperature, followed by the relative humidity and solar radiation. The most insensitive climatic factors were precipitation, wind speed and reference crop evapotranspiration. As for the yield of spring wheat, temperature, solar radiation and air relative humidity were major meteorological factors that affected in the eastern and western Inner Mongolia. Furthermore, the effect of the average minimum temperature on yield was greater than that of the average maximum temperature. The increase of temperature in the western and middle regions would reduce the spring wheat yield, while in the eastern region due to the rising temperature, the spring wheat yield increased. The increase of solar radiation in the eastern and central regions would increase the yield of spring wheat. The increased air relative humidity would make the western spring wheat yield increased and the eastern spring wheat yield decreased. Finally, the models describing combined effects of these dominant climatic factors on the maturity and yield in different regions of Inner Mongolia were used to establish geographical differences. Our findings have important implications for improving climate change impact studies and for local agricultural production to cope with ongoing climate change.

## Introduction

The predicted growth of the world population over the next few decades requires the adaptation food crops to ensure that global food supply demands can be met [1]. Approximately 21% of the world's food depends on wheat crops, which grow on 200 million hectares of cropland worldwide [2]. In an agricultural system, crop productivity varies with climatic and edaphic conditions. Climate change may negatively affect wheat yields in some major wheat production regions of the world [3]. However, these impacts still have large uncertainties and remain inconclusive in terms of their mechanisms, magnitude and spatial pattern [4]. Therefore, it is urgent to acquire a more in-depth understanding of how climate change affects crop production, ensuring global food security and making adaptation decisions for both policymakers and scientists[5,6].

Crop simulation models describing crop development and growth over time as a function of climatic factors are key tools to anticipate the effects of regional climate change on different crops and increase the understanding of crop physiology and ecology. Process-based crop models are frequently used as a scientific tool to study the impacts of management and climatic changes on crops, often with a focus on addressing global challenges, such as climate change and food and energy security [7,8]. Thus, various process-based crop models have been developed, for example, Agricultural Production Systems sIMulator (APSIM) [9], Crop Environment REsourve Synthesis (CERES) [10], WOrld FOod STudies (WOFOST) [11], Environment Policy Integrated Climate (EPIC) [12], CROP GROwth model (CROPGRO) [13], Cropping Systems simulator (CropSyst) [14], Danish simulation model (DAISY) [15], Decision Support System for Agrotechnology Transfer (DSSAT) [16], Farm ASSEssment Tool (FASSET) [17], High-Elective Resolution Modelling Emission System (HERMES) [18], and Soybean GROwth simulation model (SOYGRO) [19]. APSIM-wheat is a crop system simulation model that consists of modules that incorporate aspects of soil water, nitrogen (N), residues, and crop development. It was used to simulate aboveground and belowground growth, grain yield, water and N uptake, soil water and soil N in wheat crops. Currently, the APSIM-wheat module has been widely validated against trial data from various environments [20,21,22] and applied at sites, fields, and catchments at the continental scale [8,23,24].

The impacts of regional climate change/variability on crop growth and productivity based on the APSIM model over the past few decades in China have attracted serious concern [21,22,25,26]. These simulation results were mainly concentrated in North China, Northwest China and Northeast China, and were believed to be feasible for guiding local wheat, maize and rice production. For example, Yang et al. [22] used historical statistical crop yields and simulated crop yields from 2011–2100 in the APSIM model to quantify the impacts of changes in the northern limits of multiple cropping systems on China's crop production (maize, wheat, and rice). They found that the northern shifts of multiple cropping systems resulted in a 2.2% increase in national production of three major crops (maize, wheat, and rice) from 1981 to 2010, positively impacting China's food security. Sun et al. [26] assessed the contribution of weather and management to the annual yield variation of summer maize using the APSIM-maize model in the North China Plain. Their simulated results showed that weather factors, including sunshine hours and the diurnal temperature range during the grain fill stage, had positive effects on the maize yield. For different management practices, plant density was the most important factor that affected the maize yield.

Inner Mongolia is one of the main producing areas of spring wheat in China, and the spring wheat yield accounted for approximately 26% of the total yield of spring wheat in China from 2004 to 2012. However, few studies have been conducted to quantitatively assess the long-term impact of climate change on spring wheat production in Inner Mongolia based on the APSIM-wheat model at the regional scale. A better understanding of how spring wheat responds to regional climate change is essential for adapting the farming practices to mitigate the negative effects and even to take advantage of local climate change [27].

The objectives of the present study were to: (1) test the performance of APSIM for simulating the dynamics of the phenophases and yield of spring wheat in Inner Mongolia using robust observational evidence; (2) identify the key climatic factors limiting the phenophases and yield of spring wheat using long-term datasets; and (3) explore the combined effects of climatic factors on spring wheat phenophase and grain yield using long-term datasets in Inner Mongolia. These findings were significant for substantially improving our understanding of the response of crop production to climate change on the regional scale in China.

## Materials and methods

### Study area

Inner Mongolia is situated in the northern most part of China, sharing an international border with Mongolia and Russia. As the main producing areas of spring wheat in China, Inner Mongolia's spring wheat average yield accounted for approximately 26% of the average total production of spring wheat in China from 2004 to 2012. Its planting area accounted for approximately 30% of the spring wheat planting area in China. This region is bitterly cold in winter and warm in summer. The average annual temperature is in the range between -1°C and 10°C. The temperature in the northern part is lower, on average, than in other places. The average annual precipitation is between 50 mm and 450 mm, mostly in late summer and early autumn. The dominant climate is conducive to the growth of crops, with abundant sunshine and a hot rainy season. The frost-free period varies from 90 days to 160 days, and the temperature diurnal range is large and is conducive to dry matter accumulation of spring wheat. Spring wheat in this area is mainly rain-fed and can only be grown once per year.

**Fig 1:**
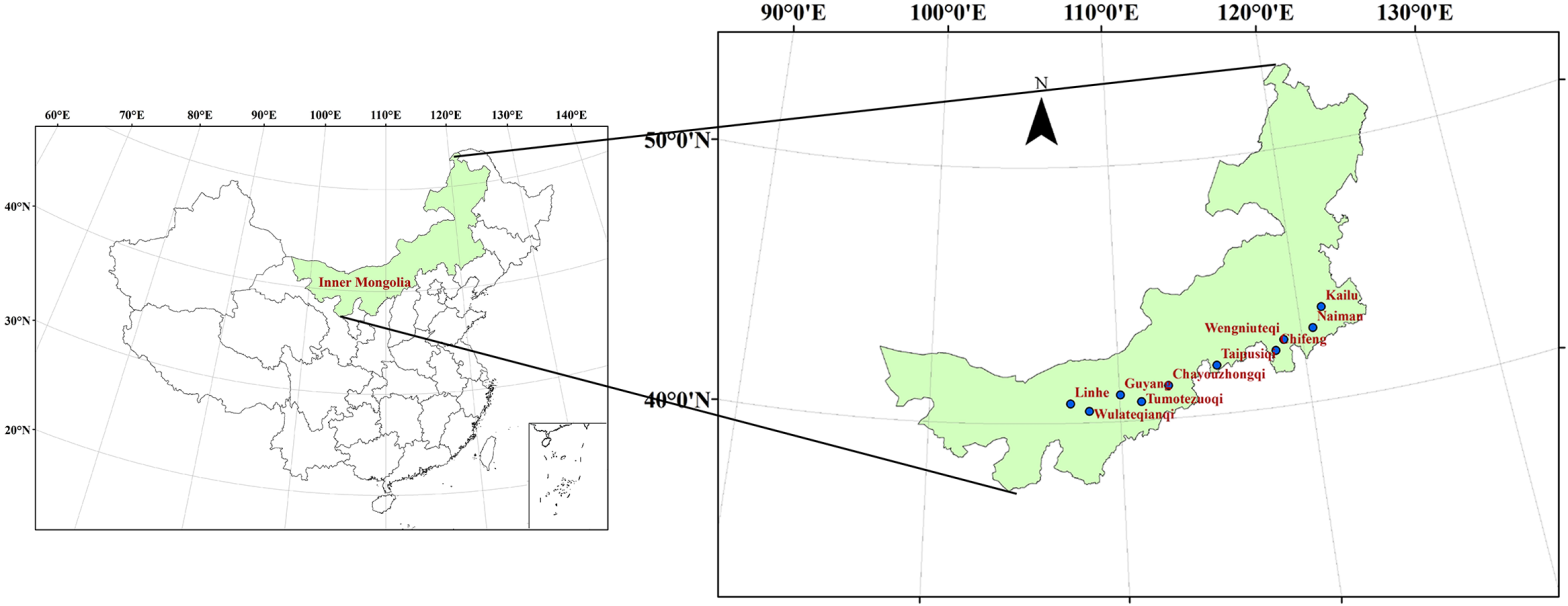
Study area location of Inner Mongolia.

Due to the large span from east to west and climatic differences, Inner Mongolia was divided into three different climate type regions, the eastern, central and western regions, in this study. Ten typical agro-meteorological experimental stations were chosen: Naiman, Chifeng, Kailu and Wengniuteqi in the eastern region; Taipusiqi, Chayouzhongqi, Guyang and Tumotezuoqi in the central region; and Linhe and Wulateqianqi in the western region. These stations covered the majority of the spring wheat planting areas. The spatial distribution of each agro-meteorological station was shown in Fig 1.

### Data sets

The meteorological data were provided by the National Meteorological Information Center, China. The crop long-term field observation datasets of spring wheat were collected from the selected 10 agro-meteorological experimental stations in Inner Mongolia, China.

### APSIM-wheat model brief introduction

APSIM, which is developed by Agricultural Production System Research Unit in Australia, is a software tool that enables sub-models (or modules) to be linked to simulate agricultural systems [28]. Sub-modules include crops, pastures, soil water, nitrogen and erosion. Here, we provide a brief overview of the important modules used in this study.

#### Climate parameters

The climate module was the basis of the APSIM model. Daily climate variables were obtained from the 10 meteorological stations between 1981 and 2014 in Inner Mongolia, and the data were provided by the National Meteorological Information Center. These daily data were organized in a database containing the following variables: the daily solar radiation, daily maximum temperature, daily minimum temperature, daily precipitation, annual average temperature, local latitude, and reference crop evapotranspiration.

#### Crop parameters

The crop parameters and long-term field observation datasets on the growth, development and yield of spring wheat were collected from the 10 agro-meteorological experimental stations in Inner Mongolia. The field observation data of crop in China have been widely recorded since the late 1970s [6]. Moreover, owing to the poor data quality from earlier years, we only collected spring wheat observation data from 1981 to 2014 in Inner Mongolia. Agro-technicians documented the yearly dates of major events, including the sowing, seedling, heading and maturity stages and the yearly crop yield for each spring wheat growth cycle at each agro-meteorological experimental station.

#### Soil parameters

The soil water module simulates the various vertical water movements in a layered soil system using a multi-layer cascading approach [29]. It updates the values of the soil water status according to the amount of daily rainfall, irrigation, water uptake by crop, soil evaporation, surface runoff and bottom drainage. The wheat crop module simulates the growth and development of a wheat crop in a daily time-step on an area basis (per square meter, not per single plant) using a CERES-wheat approach [30]. It describes the response of wheat growth and development to weather, soil water, soil nitrogen, and management practices [31] and consists of eleven phasing development stages that are determined by the accumulation of thermal time and other factors, such as vernalization, the photoperiod and N from emergence to the terminal spikelet [21]. The soil module includes the SoilN module, which describes the dynamics of both carbon and nitrogen in soil. Soil organic matter is divided into two pools (biom and hum), with the biom pool representing the more labile, soil microbial biomass and microbial products, while the hum pool comprises the rest of the soil organic matter.

The soil parameters involved in the model include soil bulk density, wilting coefficient, field water-holding capacity, saturated water content, etc. These data were from local agro-meteorological experimental stations and related research results [22,32].

### Calculation of solar radiation

The APSIM simulation model requires the input of solar radiation. However, the meteorological data acquired from the meteorological observation station were only sunshine hours. Therefore, it was necessary to calculate the solar radiation according to sunshine hour. The calculation formula was as follows [33,34]:

$$R_{n\text{s}}=0.77\times (0.25+0.5\times n/N)\times R_{a}$$ (1)

$$R_{a}=37.6\times d_{r}\times (W_{S}\times \text{sin}\varphi \times \text{sin}\delta +\text{cos}\varphi \times \text{cos}\delta \times \text{sin}W_{S})$$ (2)

$$d_{r}=1+0.033\times \text{cos}(2\pi /365\times J)$$ (3)

δ=0.409×sin(2π/365×J−1.39) (4)

$$W_{S}=\text{arccos}(-\text{tan}\varphi \times \text{tan}\delta )$$ (5)

$$N=\frac{24}{\pi }\times W_{S}$$ (6)

where *R*_*ns*_ is the daily net shortwave radiation (MJ·m^-2^·d^-1^); *R*_*a*_ is the solar radiation in a clear sky (MJ·m^-2^·d^-1^); *d*_*r*_ is the relative distance between the sun and earth;*φ* is the latitude of the station (Rad); *δ* is the solar declination angle (Rad); *W*_*s*_ is the sunrise hour angle (Rad); *J* is the day of the year; and *n* and *N* are the observed and theoretical sunshine hours under clear-sky conditions, respectively. These calculation results were verified by the observation data of local meteorological stations and was considered to be applicable in the northern China [32].

### Calculation of reference crop evapotranspiration

The APSIM simulation model requires the input of reference crop evapotranspiration (ET_0_). A large number of equations for estimating ET_0_ are available [35,36,37]. The Penman–Monteith (FAO-56 PM) equation is recommended as the standard method for estimating ET_0_ from full climate records by the Food and Agriculture Organization of the United Nations (FAO) [38,39]. In this study, the FAO-56 PM equation was used, as it incorporated both energy balance and aerodynamic theory and estimated the reference crop (height of 0.12 m, surface resistance of 70 sm^-1^ and albedo of 0.23). The computation ET_0_ followed the recommendations of the FAO [34]:

$$ET_{0}=\frac{0.408\Delta (R_{n}-G)+\gamma (900/T+273)U_{2}(e_{\text{s}}-e_{a})}{\Delta +\gamma (1+0.34U_{2})}$$ (7)

where *ET*_*0*_ is the reference crop evapotranspiration (mm·d^-1^); Δ is the slope of the saturated water-vapour pressure curve (kPa·°C^-1^); *R*_*n*_ is the net radiation at the surface (MJ·(m^2^ d)^-1^); *G* is the soil heat flux (MJ·(m^2^ d)^-1^); *γ* is the psychrometric constant (kPa·°C^-1^); *T* is the daily average temperature stably passing 0°C (°C); *U*_*2*_ is the daily average wind speed at 2 m above ground level (m·s^-1^); *e*_*s*_ is the saturation vapour pressure (kPa); *e*_*a*_ is the actual vapour pressure (kPa); and *e*_*s*_-*e*_*a*_ is the vapour pressure deficit (kPa). *R*_*n*,_ *G*, Δ and *U*_*2*_ can be calculated by the observation data of local meteorological stations.

### APSIM-wheat model accuracy evaluation

There are many evaluation indices that can be used to evaluate the accuracy or reliability of the simulation results [40]. In the present study, the following statistics were used to test the APSIM-Wheat model: the coefficient of determination between the simulated value and measured value (R^2^), consistency indicator (D), root mean square error (RMSE), mean absolute error (MAE), relative root mean square error (NRMSE) and model effectiveness (M_E_):

$$RMSE=\sqrt{\frac{\sum_{i=1}^{n}{\left(O_{i}-S_{i}\right)}^{2}}{N}}$$ (8)

$$NRMSE=\frac{RMSE}{O}\times 100\%$$ (9)

$$M_{E}=1-\frac{\sum {\left(O_{i}-S_{i}\right)}^{2}}{\sum {\left(O_{i}-O\right)}^{2}}$$ (10)

$$D=1-\frac{\sum {\left(S_{i}-O_{i}\right)}^{2}}{\sum \left(\left|S_{i}-O\right|+{\left|O_{i}-O\right|}^{2}\right)}$$ (11)

$$MAE=\frac{\sum \left|S_{i}-O_{i}\right|}{n}$$ (12)

where *RMSE* is the root mean square error between the simulated value and measured value; *O*_*i*_ is the measured value; *S*_*i*_ is the simulated value; *N* is the number of samples; *NRMSE* is the relative root mean square error between the simulated value and measured value, and the simulation has a higher accuracy when *NRMSE* is controlled within 10%; *O* is the measured average value; *M*_*E*_ is the model effectiveness. When *M*_*E*_> 0.5, the simulation results of the model are better. *D* is the consistency indicator between the simulated value and measured value. If *D* is closer to 1, the simulation result is better; and *MAE* is the mean absolute error.

### Calibration and validation of the APSIM-wheat model

The APSIM-wheat model was calibrated by using experimental data sets obtained during the 2008–2011 growing seasons in the 10 agro-meteorological experimental stations in Inner Mongolia. In this study, the trial and error method was used to calibrate the parameters of varieties of spring wheat, making the difference between the simulation value and measured value as small as possible. The performance of the APSIM-wheat model was validated with experimental data sets obtained during the 2011–2014 growing seasons in the 10 agro-meteorological experimental stations, which were not used for model calibration. The crop variables that were validated included the crop phenology (emergence and maturity) and spring wheat yield.

### Identification of key climatic factors affecting spring wheat phenophase and grain yield

There were multiple effects of climatic factors on crop growth and yield. Different factors had different effects on the growth and yield of spring wheat. To extract the dominant factors influencing the production of spring wheat in Inner Mongolia, 10 climatic factors during the growth period (April-September) from 1981 to 2014 were chosen and analysed in this paper. These climatic factors included the average daily temperature, average daily maximum temperature, average daily minimum temperature, average wind speed, average daily sunshine from sowing to maturity, total rainfall, average relative humidity, average daily soil surface temperature, total radiation and total reference crop evapotranspiration.

Grey theory is a method that is used for analysis, modelling, prediction, and decision making in a system that is "grey," which means that some of the messages are known and others are unknown [41]. The grey relation is the uncertainty associated between things or uncertainty associated between system factors and the main behavioural factors. Grey relational analysis, which is an important component of grey system theory, is based on the degree of similarity or differences between the trends of the main development factors and measurement factors [42]. In this study, the grey relational degree method was applied for identifying key meteorological factors that affected the phenophases and yield of spring wheat. The values of the phenophases and yield of spring wheat were used as reference sequences (X_o_ = (x_o_(1), x_o_(2), …, x_o_(n))), and the mean values of the climatic factors were taken as the comparison sequence (X_i_ = (x_i_(1), x_i_(2), …, x_i_(n))). The grey relational degree between the reference sequence X_0_ and comparison sequence X_i_ at the k point was calculated as follows:

$$R_{i}=\sum_{k=1}^{n=37}\xi_{i}(k)/n$$ (13)

ξi(k)=MiniMink|X0(k)−Xi(k)|+λMaxiMaxk|X0(k)−X(k)i||X0(k)−X(k)i|+λMaxiMaxk|X0(k)−X(k)i| (14)

where *R*_*i*_ is the grey relational degree. According to the value of the *R*_*i*_, sequence, it can be evaluated and sorted, and the best sequence can be determined. *N* is the total number of years; *X*_*0*_ is the reference sequence; *ξ*_*i*_*(k)* is the relational coefficient; *X*_*i*_ is the comparison sequence; *k* is the relational point; *MinMin|X*_*0*_*(k)-X*_*i*_*(k)|* is the minimum absolute difference; *MaxMax|X*_*0*_*(k)-X*_*i*_*(k)|* is the largest absolute difference; and *λ* is the resolution coefficient (generally the value is 0.5).

## Results

### Performance of the APSIM-wheat model in Inner Mongolia

**Fig 2:**
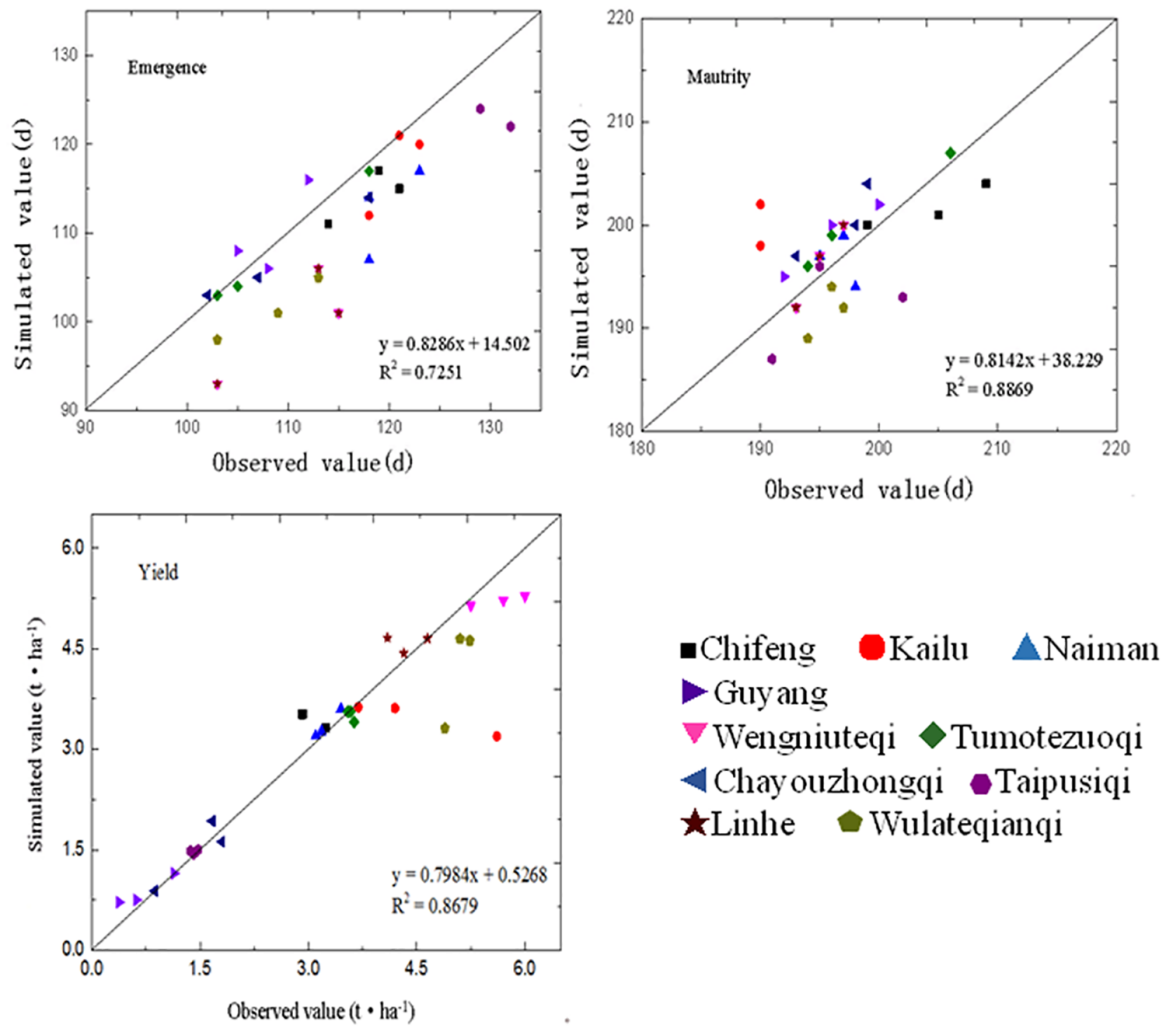
Validation results between the simulated and observed emergence DOY, maturity DOY and yields of spring wheat in Inner Mongolia.

In this study, a trial and error method for adjusting the varieties parameters of the spring wheat model in Inner Mongolia was adopted. The adjusted parameters for local phenophases of spring wheat were shown in Table 1. The day of year (DOY) during the phenophases and yield of spring wheat in the 10 sites in Inner Mongolia from 2008 to 2011 were simulated based on the APSIM-wheat model. The performance of the model was tested by comparing the measured and simulated data (Fig 2).

**Table 1:** Parameters controlling the spring wheat growth stages in Inner Mongolia.

Crop Module	Parameter type	Variety Parameter	Description	Value
Wheat	Parameter control	vern_sens	Vernalization index	1.6
		photop_sens	Photoperiod index	3.2
		tt_startgf_to_mat	Thermal time from filling to mature (°C·d)	520

For the emergence and maturity stages of spring wheat, the consistency between the simulated and measured values in the 10 sites of Inner Mongolia were good. The RMSEs during the emergence and maturity stages were 1.22d-5.49d and 1.13d-3.00d, respectively, with smaller errors. The NRMSEs were 1.06%-5.09% and 0.65%-1.45%, which were controlled within 10%. The D indices were close to 1, fluctuating between 0.49–0.99 and 0.69–1.

For the spring wheat yield, the consistency between the simulated and measured yields in the 10 sites of Inner Mongolia was also good. The RMSE of the yield fluctuated from 28.95 kg·ha^-1^ to 208.35 kg·ha^-1^. The NRMSE of the yield was 0.92%-6.4%, which was controlled within 10%. The D index was 0.85–0.95. The range of the MAE value was 41.1–410.85.

From the above analysis, we determined that the overall performance of the adjusted APSIM-wheat model was good. It more accurately simulated the growth development and yield formation of spring wheat in Inner Mongolia.

### Spatiotemporal changes of climatic variables during the growth season of spring wheat

Over the past 34 years, the daily average, maximum and minimum temperatures as well as the soil surface temperature during the growth season of spring wheat in Inner Mongolia significantly increased (P<0.01) (Table 2). However, their increase rates varied in different regions. Among them, the largest increases were found in the western wheat area, with values of 0.504°C·decade^-1^, 0.354°C·decade^-1^, 0.742°C·decade^-1^ and 0.788°C·decade^-1^. The smallest increases were found in the eastern wheat area, with values of 0.220°C·decade^-1^, 0.191°C·decade^-1^, 0.296°C·decade^-1^ and 0.330°C·decade^-1^. The total precipitation both decreased in the eastern and middle spring wheat regions, which was contrary to the increasing trend in the western wheat area. However, the decline in total precipitation was more obvious in the east (6.184 mm·decade^-1^) than that in the middle area (1.627 mm·decade^-1^). The relative humidity all decreased in three regions. Unlike the relative humidity, the total ET_0_ all increased in three regions over the past 34 years, specially with a significantly increase of 13.63 mm·decade^-1^ in the western spring wheat region. The sunshine hours showed increasing trends in the middle and western regions (P<0.01), which were opposite to the changes in the eastern region.

**Table 2:** Climate change during the growth season of spring wheat from 1981 to 2014 in Inner Mongolia.

Factor	Eastern region	Middle region	Western region
Average temperature	0.220**	0.341**	0.504**
Maximum temperature	0.191**	0.229**	0.354**
Minimum temperature	0.296**	0.525**	0.742**
Rainfall	-6.184	-1.627	3.128
Radiation	0.018	-0.105**	-0.086
Reference crop evapotranspiration	7.543	3.475	13.630**
Soil surface temperature	0.330**	0.454**	0.788**
Relative humidity	-0.466	-0.941**	-0.462**
Sunshine	0.023	-0.073**	-0.072**

Note:

*Correlation is significant at 0.05 level;

** Correlation is significant at 0.01 level

### Temporal and spatial variation of the spring wheat potential yield in Inner Mongolia

**Fig 3:**
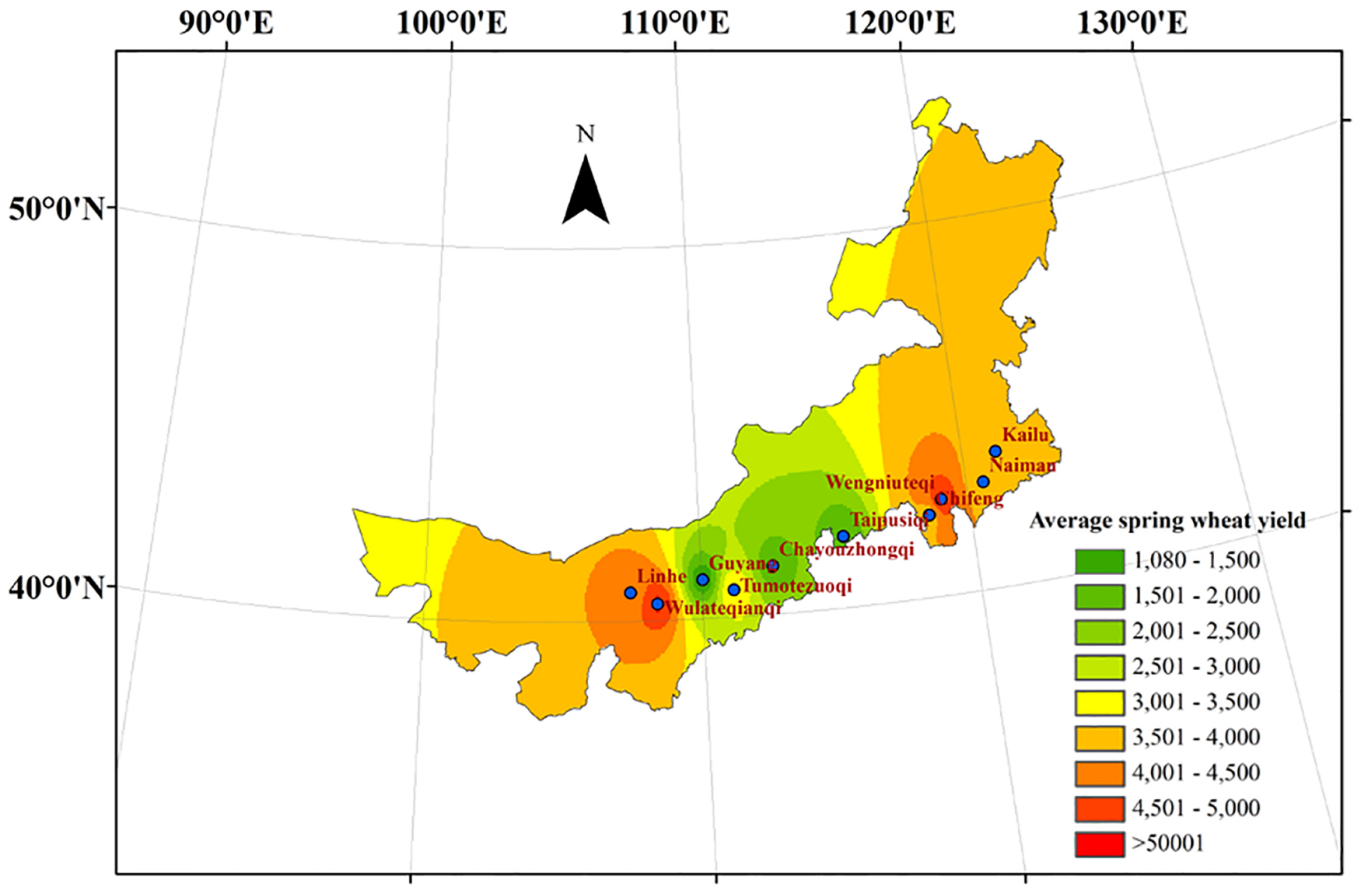
Average spatial distribution of spring wheat yield from 1981 to 2014 in Inner Mongolia (Kg·ha^-1^).

The simulated spring wheat potential yield from 1981 to 2014 in Inner Mongolia showed an overall decreasing trend, with an average of 3564 kg·ha^-1^. The highest potential yield and lowest yield were found from 1981 to 1990 (3700 kg·ha^-1^) and from 2001 to 2014 (3371 kg·ha^-1^), respectively. The spatial distribution of the spring wheat yield from 1981 to 2014 in Inner Mongolia was shown in Fig 3. The regional differences in the yields were significant. The yield of spring wheat displayed a gradually increasing trend from the middle to the eastern and western regions, fluctuating from 1733 Kg·ha^-1^ to 4361 Kg·ha^-1^. The highest yields were found in Wengniuteqi (the eastern region) and Wulateqianqi (the western region), with the values of 5024 Kg·ha^-1^ and 5094 Kg·ha^-1^, respectively. The minimum yield appeared in Guyang (the middle region), with a value of 1074 Kg·ha^-1^. The maximum yield appeared in Wulateqianqi, followed by Wengniuteqi, Linhe, Kailu, Chifeng, Naiman, Tumotezuoqi, Chayouzhongqi, Taipusiqi and Guyang. There were significant differences among the regions. The spring wheat yield was the highest in Wengniuteqi (the eastern region). In the middle region, Tumotezuoqi had the highest yield, which was far higher than that of the other stations. However, there was little difference found, with a higher yield, in Wulateqianqi than in Linhe.

In the three areas of Inner Mongolia, the simulated western spring wheat region had the highest yield over the past 34 years, with an average of 4069 Kg·ha^-1^. On the contrary, the simulated middle spring wheat region had the lowest yield, with an average of 1945 Kg·ha^-1^. These results were consistent with the actual production of spring wheat in Inner Mongolia. The natural environment and resources in western Inner Mongolia, such as fertile soil, good infrastructure, enough sunshine and high accumulation temperature, were conducive to the growth of spring wheat. As far as Northeast Inner Mongolia was concerned, it was a dryland farming area, with fertile soil. Deep snow and low temperatures in spring were suitable for wheat emergence and panicle differentiation.

### Key climatic factors affecting local spring wheat production

**Fig 4:**
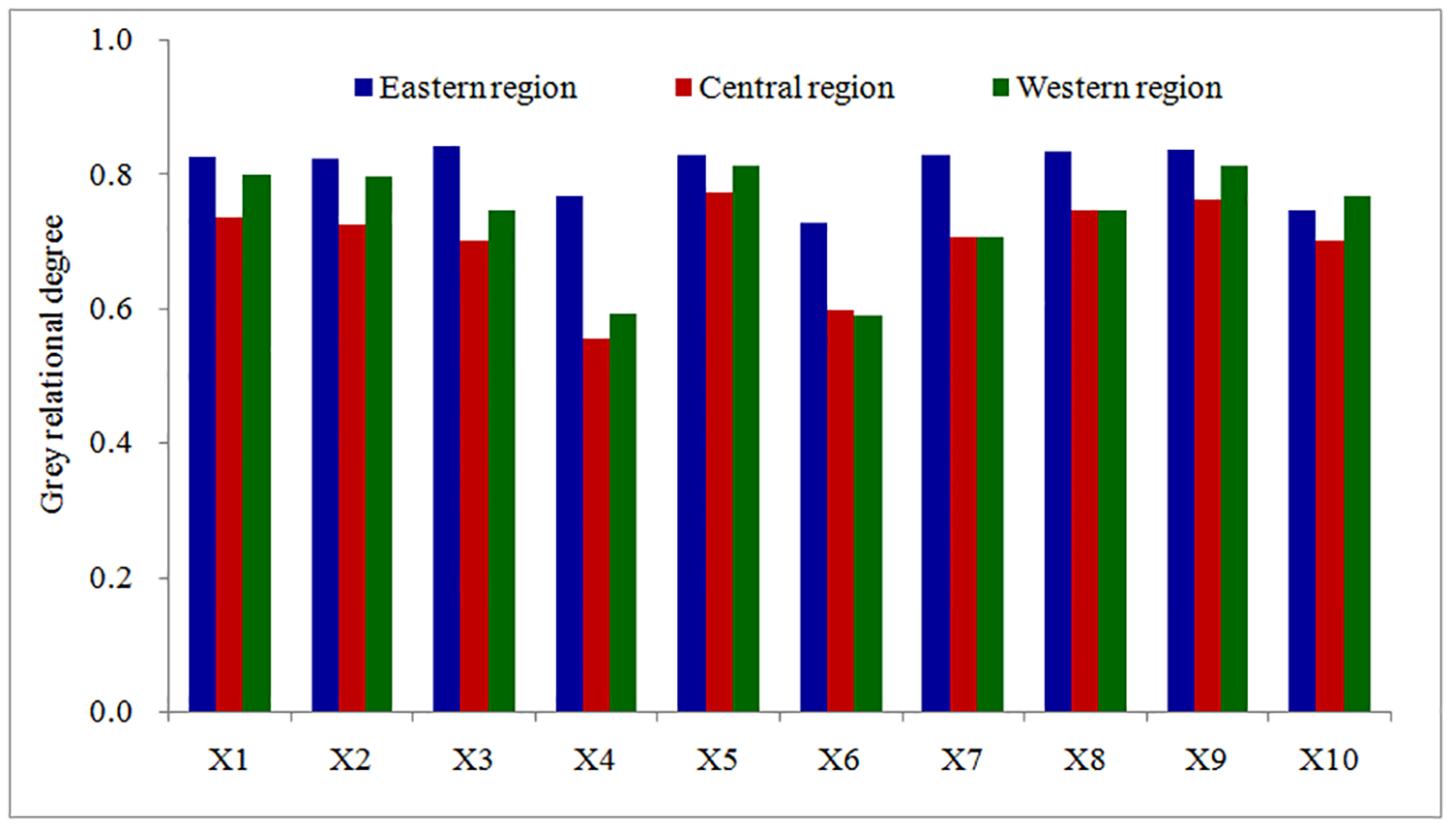
Grey correlation degree between spring wheat yield and key climatic factors from sowing to maturity in Inner Mongolia. (1) X1 is the average daily temperature; (2) X2 is the average daily maximum temperature; (3) X3 is the average daily minimum temperature; (4) X4 is the average wind speed; (5) X5 is the average daily sunshine from sowing to maturity; (6) X6 is the total rainfall; (7) X7 is the average relative humidity; (8) X8 is the average daily soil surface temperature; (9) X9 is the total radiation; and (10) X10 is the total reference evapotranspiration.

The main climatic factors influencing the production of spring wheat in Inner Mongolia were the temperatures and solar radiation. However, the optimum climatic factors affecting the spring wheat yield in the different regions of Inner Mongolia were different (Fig 4). In the eastern area, the main meteorological factors affecting the spring wheat yield were the average temperature and average minimum temperature during the growth period, followed by the total radiation, average soil temperature and average maximum temperature. If the average temperature, average minimum temperature, total radiation, soil temperature and average maximum temperature increased by 1°C, the average yield of spring wheat would increase by 303 Kg·ha^-1^, 270 Kg·ha^-1^, 250 Kg·ha^-1^, 235 Kg·ha^-1^ and 231 Kg·ha^-1^, respectively. In the western area, the optimal factors affecting the spring wheat yield were the average soil surface temperature, average temperature and average maximum temperature, followed by the average minimum temperature, total ET_0_ and relative humidity. The yield of spring wheat obviously decreased with the increase of temperature. If the average maximum temperature, average temperature increased by 1°C, the average yield of spring wheat would reduce 198 Kg·ha^-1^ and 162 Kg·ha^-1^. However, changes in the spring wheat potential yield caused by the average precipitation and wind speed were not obvious. In the middle area, the spring wheat yield was the most sensitive to solar radiation and average temperature, followed by the average minimum temperature, average maximum temperature and average soil temperature. If the total radiation increased by 1 MJ•m^-2^, the yield of spring wheat would increase by 154 Kg·ha^-1^. If the average temperature, average minimum temperature, average maximum temperature and average soil temperature increased by 1°C, the average yield of spring wheat would decrease by 107 Kg·ha^-1^, 106 Kg·ha^-1^, 79 Kg·ha^-1^ and 69 Kg·ha^-1^, respectively. However, it was the least sensitive to the average wind speed and total precipitation.

**Fig 5:**
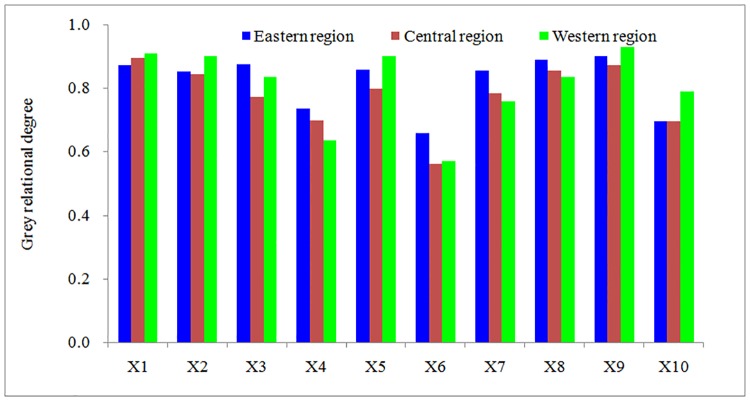
Grey correlation degree between the maturity DOY of spring wheat and key climatic factors from sowing to maturity in Inner Mongolia. (1) X1 is the average daily temperature; (2) X2 is the average daily maximum temperature; (3) X3 is the average daily minimum temperature; (4) X4 is the average wind speed; (5) X5 is the average daily sunshine from sowing to maturity; (6) X6 is the total rainfall; (7) X7 is the average relative humidity; (8) X8 is the average daily soil surface temperature; (9) X9 is the total radiation; and (10) X10 is the total reference evapotranspiration.

In terms of maturity, the key climatic factors influencing the maturity DOY of spring wheat in the three regions of Inner Mongolia were mainly the temperature, relative humidity and solar radiation from sowing to maturity (Fig 5). However, the sensitivities of the maturity DOY of spring wheat to different climatic factors in these three regions were different. In the eastern area, the most sensitive factor was the average relative humidity, followed by the average daily soil surface temperature, total radiation, average daily minimum temperature, average daily temperature, average daily maximum temperature, average wind speed, total rainfall and ET_0_ from sowing to maturity. In the middle area, the most sensitive factor was the average daily temperature, followed by the average daily soil surface temperature, total radiation, average daily maximum temperature, average relative humidity, average daily minimum temperature, total ET_0_, average wind speed and total rainfall. In the western area, the most sensitive factor was the average daily temperature, followed by the total radiation, average daily soil surface temperature, average daily maximum temperature, average daily minimum temperature, average relative humidity, total ET_0_, average wind speed and total rainfall. With the increase of temperature, the maturity DOY of spring wheat was significantly advanced. These results can provide technical support for analysing the limiting factors of spring wheat growth and yield formation in the future wheat-producing areas in Inner Mongolia.

### Combined effects of different climatic factors on the phenophases and yield of spring wheat

Based on the response analysis of the phenophases and yield of local spring wheat to climatic factors, the maturity and yield of spring wheat were found to be more sensitive to climatic factors. Firstly, the dominant climatic factors affecting the phenophases and yield of local spring wheat were extracted by the grey relational analysis method. Then, combined effects of these dominant climatic factors on the maturity and yield in different regions of Inner Mongolia were explored (Table 3).

**Table 3:** Combined effects of key climatic factors on the maturity and yield in the different regions of Inner Mongolia.

Region	Regression model
Eastern Inner Mongolia	*Y*_*M*_ = 287.703–1.341*T*-1.936*T*_*MAX*_-1.066*T*_*MIN*_-0.007*T*_*S*_
	*Y*_*C*_ = -8327.337–280.791*T*+100.066*T*_*MAX*_+184.857*T*_*MIN*_+255.663*R*+38.061*H*+252.351*T*_*S*_
Western Inner Mongolia	*Y*_*M*_ = 257.292–0.633*T*-1.068*T*_*MAX*_-2.336*T*_*MIN*_-0.007*ET*_*0*_+0.504*T*_*S*_
	*Y*_*C*_ = 9468.853–83.909*T*-156.964*T*_*MAX*_+42.269*T*_*MIN*_+6.14*H*
Central Inner Mongolia	*Y*_*M*_ = 281.807+10.252*T*-5.07*T*_*MIN*_+0.155*R*-0.252*T*_*S*_-7.73*T*_*MAX*_
	*Y*_*C*_ = 2358.603+827.796*T*-473.832*T*_*MAX*_-380.16*T*_*MIN*_+74.823*R*-54.685*T*_*S*_

Note: *Y*_*M*_ is the day of year (DOY) during the stage of maturity; *T* is the average daily temperature from sowing to maturity (April-September); *T*_*MAX*_ is the average daily maximum temperature from sowing to maturity; *T*_*MIN*_ is the average daily minimum temperature from sowing to maturity; *T*_*S*_ is the average daily soil surface temperature from sowing to maturity; *Y*_*C*_ is the spring wheat yield;; *R* is the total radiation from sowing to maturity; *H* is the average relative humidity from sowing to maturity; and *ET*_*0*_ is the total reference evapotranspiration from sowing to maturity.

## Discussion

### Effect of different climatic variables on spring wheat production

Temperature plays a dominant role in determining the duration of crop developmental phases [43]. Recent researches have progressed worldwide in response to temperature increases [44,45]. Our data also showed that the temperatures were the dominant factors influencing the different phenological stages of spring wheat in Inner Mongolia. Increasing temperatures accelerated the physiological growth of spring wheat. In turn, these changes eventually resulted in an overall reduction of durations of vegetative growth, reproductive growth and the whole growing season for spring wheat. In particular, compared with the air temperature, the soil temperature had much more of a direct effect on the germination and emergence of seeds. The higher soil temperature accelerated crop growth and development. The results of this study further confirmed that the soil surface temperature was one of the dominant factors for the spring wheat phenophases in Inner Mongolia.

Consistent with previous studies [46], variations in precipitation did not exert significant impacts on the spring wheat yield compared to the other factors in our study. This was not surprising since the drought-resistant ability of spring wheat was very strong and the precipitation basically met the water demand for spring wheat in semi-arid regions of China, such as Inner Mongolia. Another possible explanation was offered by the variability differences among the variables. In our dataset, the transformed precipitation from May to September varied by ±21%, ±29% and ±22% (relative SD), while the spring wheat yield varied by ±9%, ±6% and ±9% in the eastern, western and middle areas, respectively.

Spring wheat is a heliophile. Its growth morphogenesis and yield have strong reactions that are dependent on light intensity and sunshine. Light affects not only spring wheat growth but also its assimilation distribution. Long sunshine hours are conducive to spring wheat. In our study, we found that solar radiation during the whole growth in Inner Mongolia was both sensitive to the phenophase and yield of spring wheat. Although the solar radiation decreased with fluctuation over the past 34 years in this region, radiation could still ensure the growth of spring wheat in general. This was because northern China had more dry and sunny days and a greater total amount of solar radiation compared with southern China [6]. In addition, decreased radiation meant lower daytime temperatures and could mitigate the effects of high temperature stress on crop production to some extent. In addition, other agricultural practices such as adjusting sowing date and plant density could also reduce the negative impact of local climate change on spring wheat.

### Limitations and implications of the study

Evaluating the impacts of climate change on agriculture production depends largely on the performance of process-based crop models [5]. Therefore, these crop models need to be parameterized, calibrated and validated using experimental trials before typical development [47]. In common with process-based simulation and other empirical studies, it must be noted that several areas of limitation and uncertainty may outweigh our final results. Firstly, uncertainties in the APSIM model outputs resulted from variations in the cultivar parameters and crop observation datasets. The cultivar parameters were normally determined by a literature review or expert opinion or were calibrated against trial data. As certain parameters could vary from one climatic condition to another, the uncertainties derived from the cultivar parameters should always be considered when making decisions based on simulated results. Hence, the values of the cultivar parameters, especially the most influential ones, should be carefully determined; otherwise, unreliable model results were likely [48]. In addition, if the observed spring wheat development period and yield were accurate and representative (or not), they would directly affect the calibration and validation of the APSIM-wheat model. However, most of the observed crop data in China were still based on manual observations in Chinese agricultural meteorology experiment stations [37]. These measures contained some observation errors, which were not considered in this study, and could outweigh the simulation results. Secondly, the statistical models established according to the phenophases and yield of spring wheat and climatic factors would still cause some regional deviations because the actual relationships would vary due to the different climatic zones, latitude and longitude, topography, and soil types [49]. It is well known that all regional empirical/statistical models are scale-dependent and cannot reliably predict responses at sub-regional scales. Therefore, these models require further verification and an in-depth analysis at sub-regional scales, with the continuous improvement of data in the future. Thirdly, we only considered the main meteorological factors during the spring wheat growth season and did not cover other important factors, such as extreme events, fertilizer input, water availability and pesticide use, which could all influence the agricultural production capacity and might cause additional yield losses. Furthermore, the climate parameters and their seasonal average in this study would increase the stress of the environment change impact. With the gradual accumulation and advantages of high temporal and spatial resolution crop and climate data, further research should pay more attention on how to reduce these large uncertainties in the response of spring wheat to climate change in Inner Mongolia.

## Conclusion

This paper investigated the combined effects of climatic factors on spring wheat phenophase and grain yield over the past three decades in Inner Mongolia based on the APSIM-wheat model. From comprehensive model-observation comparisons, it was concluded that the APSIM-wheat model was able to accurately simulate the interactions between spring wheat and climatic constraints in the planting areas of spring wheat in Inner Mongolia. Our results revealed a general yield reduction of spring wheat in response to the pronounced climate warming over the past three decades. The regional differences in yields were significant. The maximum potential yield of spring wheat was found in the western region. However, the middle region had the minimum potential yield. The air temperature and soil surface temperature were the optimum climatic factors affecting the spring wheat phenophases in Inner Mongolia, followed by relative humidity and solar radiation. The most insensitive meteorological factors affecting the spring wheat phenophases were precipitation, wind speed and reference crop evapotranspiration. Temperature, solar radiation and air relative humidity were major climatic factors that affected the yields of spring wheat in eastern and western Inner Mongolia. Furthermore, the effect of the average minimum temperature on yield was greater than that of the average maximum temperature. The temperature increases in the western and middle regions had negative impacts on spring wheat yield, while in the eastern region due to the rising temperature, the spring wheat yield increased. The solar radiation increase in the eastern and central regions had positive impacts on the yield of spring wheat. The increased air relative humidity would make the western spring wheat yield increased and the eastern spring wheat yield decreased. Finally, the models describing combined effects of these dominant climatic factors on the maturity and yield in different regions of Inner Mongolia were used to establish geographical differences.

In summary, the results of this study have important implications for the improvement of climate change impact studies for agricultural production in China when attempting to mitigate the negative effects of climate change. In addition, future studies should also consider local cultivar-specific responses to extreme climate change based on field and chamber control studies. Furthermore, attempts should be made to simultaneously modify the climate change module and wheat cultivar change module of APSIM for more accurate applications to local conditions.

## Supporting information

S1 File (ZIP)

S2 File (PDF)

S1 Table Basic information of typical meteorological station in Inner Mongolia. (DOCX)
