# Supplementary figures and images for: Assessing the combined effects of climatic factors on spring wheat phenophase and grain yield in Inner Mongolia, China
Source: PLoS One. 2017 Nov 3;12(11):e0185690. doi: 10.1371/journal.pone.0185690 (PMC5669425; doi:10.1371/journal.pone.0185690)

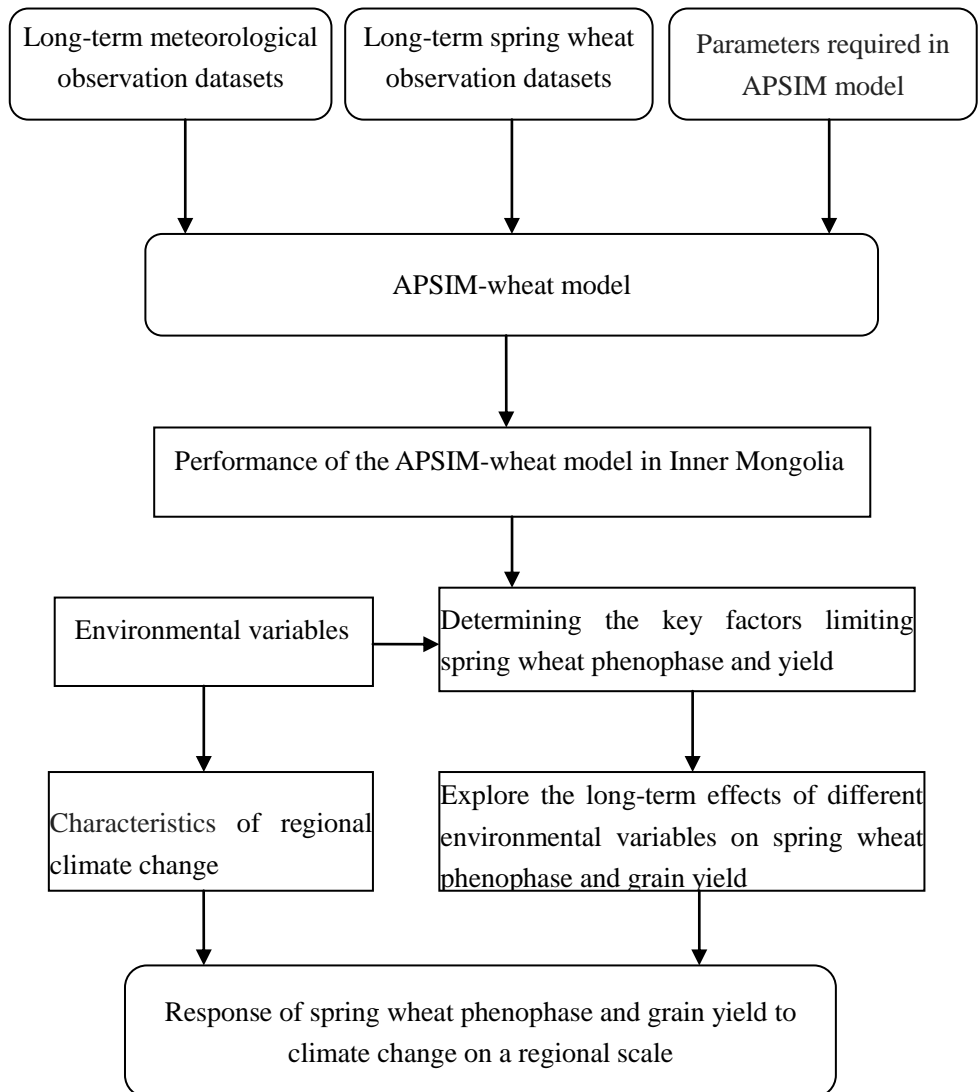

Supplement: S2 File — (PDF) [file pone.0185690.s002.pdf]
