# Supplementary material for: Assessing the combined effects of climatic factors on spring wheat phenophase and grain yield in Inner Mongolia, China
Source: PLoS One. 2017 Nov 3;12(11):e0185690. doi: 10.1371/journal.pone.0185690 (PMC5669425; doi:10.1371/journal.pone.0185690)
Supplement: S1 Table — (DOCX) [file pone.0185690.s003.docx]

**S1 Table. Basic information of typical meteorological station in Inner Mongolia**

| Region | Station | Latitude (°) | Longitude (°) |
| --- | --- | --- | --- |
| Eastern Inner Mongolia | Kailu | 43.60 | 121.28 |
|  | Naiman | 42.85 | 120.65 |
|  | Wengniuteqi | 42.56 | 119.01 |
|  | Chifeng | 42.18 | 118.50 |
| Central Inner Mongolia | Taipusiqi | 41.89 | 115.27 |
|  | Chayouzhongqi | 41.28 | 112.62 |
|  | Guyang | 41.03 | 110.05 |
|  | Tumotezuoqi | 40.72 | 111.15 |
| Western Inner Mongolia | Linhe | 40.75 | 107.42 |
|  | Wulateqianqi | 40.44 | 108.39 |
